# Food allergy and anaphylaxis – 2042. High rates of egg reactivity in infants with eczema randomised to receive egg under 6 months of age

**DOI:** 10.1186/1939-4551-6-S1-P127

**Published:** 2013-04-23

**Authors:** Jessica Metcalfe, Debra Palmer, Susan Prescott

**Affiliations:** 1 School of Paediatrics and Child Health, University of Western Australia, Australia

## Background

Egg allergy is highly prevalent in children with eczema, however there is little known about the establishment of sensitization and clinical reactivity in infants under 6 months of age.

## Methods

49 infants between the ages of 4-5 months were randomised to receive pasteurized raw egg powder as the intervention for a randomised controlled trial. All infants had moderate to severe eczema with SCORAD score >15, and had no known prior exposure to egg. Data was collected on any allergic reactions to the egg powder, along with background information on the infants and their families including allergic history, health, diet and environment. The infants consumed ¼ to 1 teaspoon of pasteurized raw egg powder daily for 3 to 4 months unless an allergic reaction to this study powder was confirmed.

## Results

Positive allergic reactions occurred in 16/49 (32.6%) infants who received the pasteurized raw egg powder. Skin reactions were the most common symptom with skin rashes (peri-oral redness and exacerbation of eczema on face or body) in 15/16 (93.7%) infants, as well as urticaria and/or angioedema in 11/16 (68.7%) each. Of less frequency were gastrointestinal (GI) symptoms (usually vomiting): 5/16 (31.2%), and respiratory symptoms: 2/16 (12.5%). Severe reactions were observed in 2/16 infants (18.75%) with one infant with FPIES and one case of anaphylaxis. Reactions occurred within 60 minutes in 12/16 (75%) infants, with seven reactions (43.7%) presenting immediately within 5 minutes. Later onset between 1-3 hours occurred in 4/16 infants (25%), with two cases experiencing severe GI symptoms. Delayed reactions after three hours of ingestion were not observed in this group. There were no differences in background characteristics of the infants who reacted compared to those who did not react to the egg powder, including family allergic history, infant medical history, feeding practices, or exposure to other children.

## Conclusions

Infants with eczema under 6 months of age are at high risk of allergic reactions with their ‘first’ introduction of egg, including severe symptoms of FPIES and anaphylaxis. This highlights the need to understand the much earlier events leading to food sensitisation.

